# Two acyclic imides: 3-bromo-*N*-(3-bromo­benzo­yl)-*N*-(pyridin-2-yl)benzamide and 3-bromo-*N*-(3-bromo­benzo­yl)-*N*-(pyrimidin-2-yl)benzamide

**DOI:** 10.1107/S2056989020014413

**Published:** 2020-11-03

**Authors:** Féilim Desmond, John F. Gallagher, Niall Hehir

**Affiliations:** a School of Chemical Sciences, Dublin City University, Dublin 9, Ireland

**Keywords:** bromine, crystal structure, imide, halogen bonding, hydrogen bonding

## Abstract

The title compounds, C_19_H_12_Br_2_N_2_O_2_ and C_18_H_11_Br_2_N_3_O_2_, were synthesized in good yields from condensation reactions of 3-bromo­benzoyl chloride with 2-amino­pyridine or 2-amino­pyrimidine using standard condensation reaction conditions and subsequent column chromatography.

## Abstract 2

The title acyclic *meta*-bromo substituted imide derivatives were synthesized in good yields from condensation reactions of 3-bromo­benzoyl chloride with 2-amino­pyridine or 2-amino­pyrimidine using standard condensation reaction conditions and subsequent column chromatography.

## Chemical context   

Acyclic imide chemistry, as *R*CON(*R*′)CO*R*, (where *R*,*R*′ are aryl or alkyl groups) has developed over the past 130 years from condensation reactions of benzoyl chlorides with amino-aromatics such as 2-amino­pyridines or 2-amino­pyrimidines (Marckwald, 1894 ▸; Tschitschibabin & Bylinkin, 1922 ▸; Huntress & Walter, 1948 ▸). From these reactions, a mixture of the benzamide and acyclic imide is usually obtained, with the relative yields of each component dependent on the starting materials and reaction conditions. The imides can also be synthesized directly from a benzamide starting material. The presence of an *ortho*-N in the benzamide heteroaromatic ring is an important feature needed to obtain the imide derivative in good yields (Mocilac *et al.*, 2010 ▸, 2012 ▸; Khavasi & Tehrani, 2013 ▸).
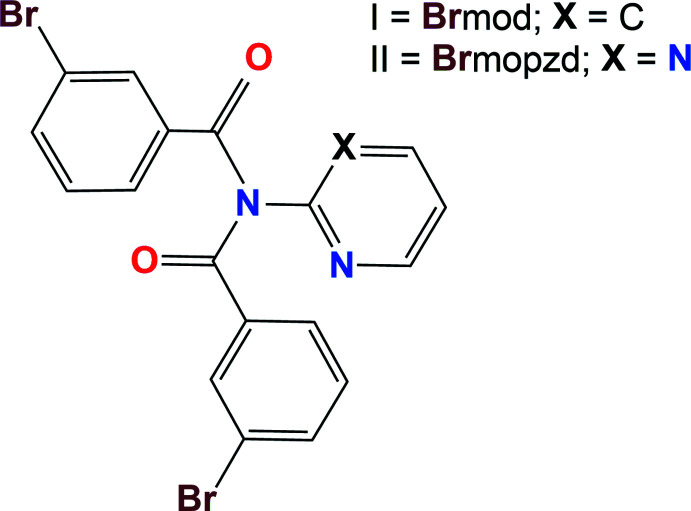


Several *R*CON(*R*′)CO*R* structures have been reported (Groom *et al.*, 2016 ▸) and derive mostly from either *R*′ = benzene (Baell *et al.*, 2001 ▸) or *R*′ = pyridine or pyrimidine groups (Gallagher *et al.*, 2009*a* ▸,*b* ▸; Mocilac *et al.*, 2018 ▸). Related imide structures include the halo­imide *N*-(2,4-di­chloro­phen­yl)-2-methyl-*N*-(2-nitro­benzo­yl)benzamide (Saeed *et al.*, 2010 ▸) or CSD (Groom *et al.*, 2016 ▸) refcode LAKXIG. LAKXIG adopts an open imide or *anti* conformation with respect to the benzoyl rings and is notable for having three different *ortho*-benzene substituents. QADPER or *N*-(3-meth­oxy­phen­yl)-*N*-(3-meth­oxy­benzo­yl)benzamide, a meth­oxy­imide derivative has been studied in the design and synthesis of type-III mimetics of the ω-conotoxin GVIA polypeptide (Baell *et al.*, 2001 ▸) and is similar in structure to several haloaromatic imides (Gallagher *et al.*, 2009*a* ▸,*b* ▸; Mocilac *et al.*, 2018 ▸; Shukla *et al.*, 2018 ▸). Kohmoto *et al.*, (2001 ▸) have described a series of 9-anthryl-*N*-(naphthyl­carbon­yl)carboxamides having the *syn-*type structure and has been used in photo­cyclo­addition reactions. Masu *et al.*, (2005 ▸) expanded on this research into di­imides to develop foldamer chemistry with the central moiety in these imide structures usually being an alkyl aromatic group.

In recent research on macrocyclic imides, we and others have noted the role of the imide hinge in the development of macrocyclic imides (Evans & Gale, 2004 ▸; Mocilac & Gallagher, 2013 ▸). Both *syn* and *anti* types of acyclic imide conformation have been observed in the macrocycles. It has been noted how this affects the formation of both trezimide and tennimide macrocycles and with the *syn* conformation essential for trezimide formation (Mocilac & Gallagher, 2013 ▸). Further studies are needed to demonstrate the ease with which the two distinct conformations can inter­convert in acyclic imides.

## Structural commentary   

**Figure 1:**
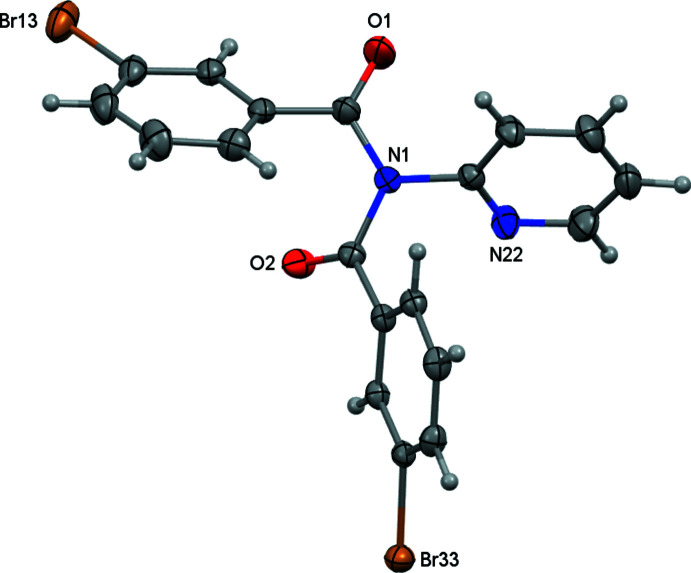
An *ORTEP* view of (I) with the atomic numbering scheme. Displacement ellipsoids are drawn at the 30% probability level.

**Figure 3:**
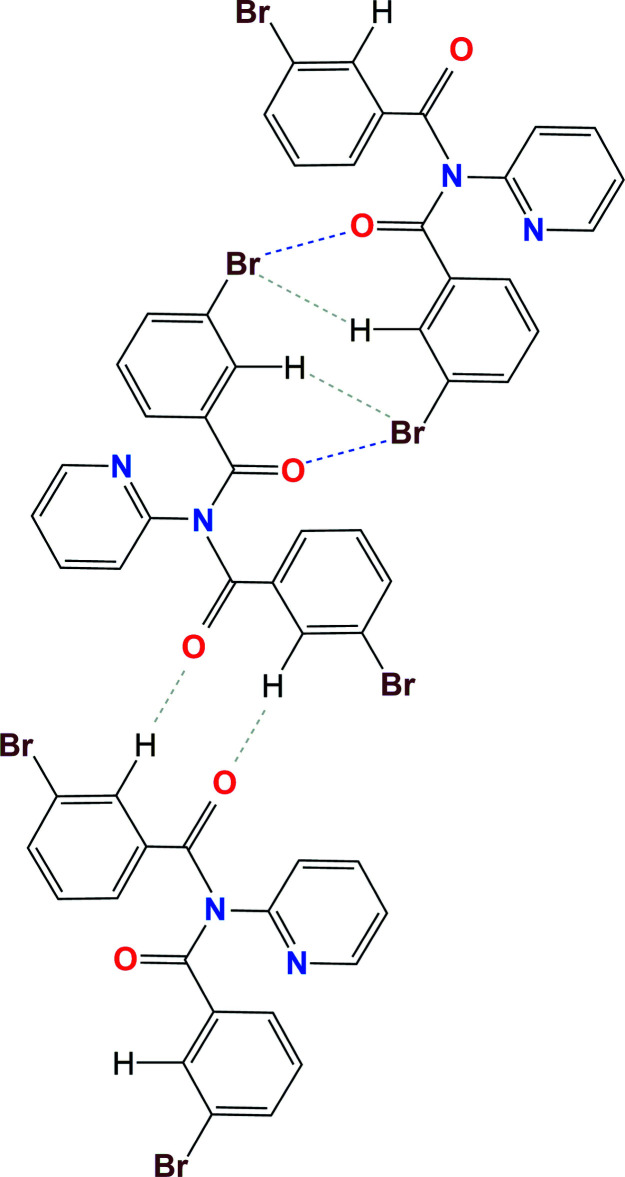
A schematic diagram of the hydrogen- and halogen-bonding inter­actions in the crystal structure of (I).

From the condensation reaction of *meta*-BrC_6_H_4_COCl with 2-amino­pyridine and 2-amino­pyrimidine, the benzamide and imide products were obtained and separated by standard column chromatography for each reaction. Using 2-amino­pyridine, **Brmo** and **Brmod**, (I) were obtained and for 2-amino­pyrimidine, **Brmopz** and **Brmopzd**, (II) were isolated. **Brmo** and **Brmopz** are the (1:1) benzamide products, whereas **Brmod**, (I) and **Brmopzd**, (II) are the (2:1) acyclic imides. Both (I) and (II) (Figs. 1 ▸–2 ▸) adopt similar mol­ecular structures to the majority of published structures (Groom *et al.*, 2016 ▸; Gallagher *et al.*, 2009*a* ▸,*b* ▸) but they differ in their supra­molecular features (Figs. 3 ▸–7 ▸ ▸ ▸ ▸). Both mol­ecules lack strong donor groups (no amide group as in the benzamides; Donnelly *et al.*, 2008 ▸) but have strong acceptors such as O=C and *N*-heteroaromatic rings that are able to participate in many weaker inter­molecular inter­actions in their crystal structures, not to mention potential π-ring aromatic stacking and C—H⋯π inter­actions (Martinez & Iverson, 2012 ▸; Nishio, 2004 ▸).

A comparison of acyclic imides and their key torsion angles demonstrates the range of angles observed and the key differences between the *syn* (carbonyl O⋯O separations of ∼4.5 Å) and *anti* conformations (O⋯O separations of ∼3.7 Å) in crystal structures (Groom *et al.*, 2016 ▸). In (I) the O1⋯O2 distance is 3.871 (3) Å and the O1=C1⋯C2=O2 torsion angle is −109.3 (5)° compared to an O1⋯O2 = 3.646 (5) Å distance and an O1—C1⋯C2=O2 torsion angle of −96.6 (5)° in (II). We have also previously used the *cisoid* and *transoid* terminology for the disposition of the two C=O groups; this is used to describe the orientation and direction of the C=O groups/aromatic rings with respect to one another (Mocilac *et al.*, 2018 ▸).

## Supra­molecular features   

The prevalent *anti*-conformation imide structural type is demonstrated in the structures of both (I) and (II) and is similar to the mol­ecular structures of the *ortho*-F (SOLSUI) and *meta*-F (DOKXOR) imide structures (Gallagher *et al.*, 2009*a* ▸,*b* ▸), the chloro- and methyl-imides (Mocilac *et al.*, 2018 ▸) and two benzene relatives (Shukla *et al.*, 2018 ▸). This contrasts with the *syn* type as observed in the crystal structure of **Mood**, a 2-methyl­benzoyl imide (Mocilac *et al.*, 2018 ▸) and the four recently described SEYSUN/SEYTIC/SEYTOI/SEYTUO structures (Shukla *et al.*, 2018 ▸). A key difference between these structures is the central *N*-pyridine ring in **Mood** (Gallagher *et al.*, 2009*a* ▸,*b* ▸) and *N*-benzene rings in the SEYSUN-type structures (Shukla *et al.*, 2018 ▸).

In (I), the **Brmod** mol­ecules aggregate as dimers in a cyclical arrangement using the C32—H32⋯Br33^ii^ and C2=O2⋯Br33^ii^ inter­actions with the 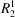
(6) motif. Two of these combine to form the centrosymmetric 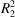
(12) motif as formed by the flanking C=O⋯Br—C halogen-bonding inter­actions (Figs. 3 ▸, 5 ▸ and 6 ▸). The hydrogen bonding as H32⋯Br33^ii^ has *N*_C_ = 0.986 (Table 1 ▸) where *N*_C_ is the ratio of contact distance/sum of contact radii using data from Bondi (Bondi, 1965 ▸; Spek, 2020 ▸). The halogen-bonding geometric details are Br33⋯O2^ii^ = 3.287 Å (symmetry code ii; Table 1 ▸) or *N*_C_ = 0.975 with C33—Br33⋯O2^ii^ = 156.85 (9)° and Br33⋯(O2=C2)^ii^ = 134.11 (19)° angles. Centrosymmetric C—H⋯O hydrogen-bonding inter­actions as 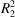
(10) link dimers into zigzag chains along the *b*-axis direction, whereas weak C—H⋯N inter­actions link chains into ruffled sheets parallel with the (100) plane (Table 2 ▸).

**Figure 4:**
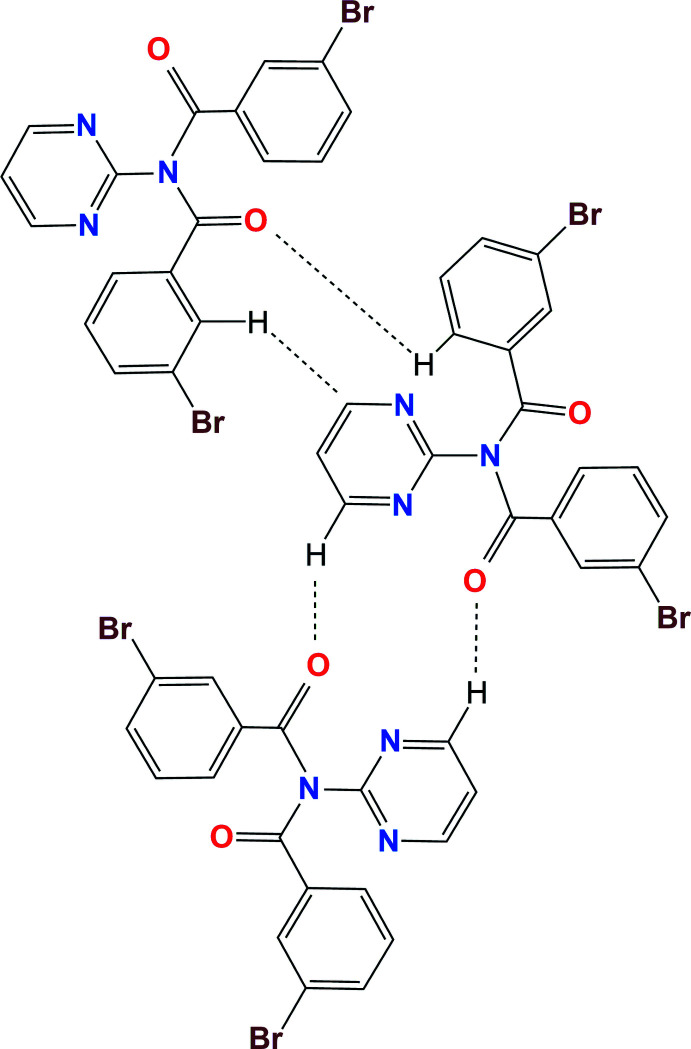
A schematic diagram of the main inter­molecular inter­actions in the crystal structure of (II).

In (II), the **Brmopzd** mol­ecules aggregate by weak inter­molecular inter­actions, as two C—H⋯O, two C—H⋯π(arene) and a C—Br⋯π(arene) contact per mol­ecule, to generate a 3D structure (Figs. 4 ▸ and 7 ▸). The C36—H36⋯O2^ii^ and C25⋯(H12—C25)^ii^ inter­actions combine together in the aggregation of a pair of tightly bound mol­ecules with graph-set 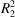
(15), while the remaining C23—H23⋯O1^i^ hydrogen bond results in the formation of centrosymmetric dimers in tandem with π–π stacking between the pyrimidyl rings, with shortest contact distances for N22⋯C23^i^ = 3.429 (6) Å and N22⋯C24^i^ = 3.464 (7) Å. The C13—Br13⋯π(arene)^iv^ contact [symmetry code: (iv) 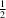 + *x*, 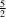 − *y*, *z*] has a Br13⋯C15^iv^ distance of 3.550 (6) Å and C13—Br13⋯C15^iv^ = 149.44 (16)°, where C15^iv^ represents the closest Br⋯C contact on the arene ring. The N atoms (two pyrimidyl or tertiary amine N) do not participate in inter­molecular inter­actions and the shortest contact is N26⋯H24^v^ = 2.76 Å [symmetry code: (v) 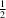 − *x*, 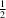 + *y*, 2 − *z*) (Spek, 2020 ▸).

## Database survey   

A literature search for acyclic imides provides several 2-amino­pyridine structures of which DOKXOR a *meta*-F benzene derivative (Gallagher *et al.*, 2009*a* ▸) and CIJPET a *meta*-Cl derivative (Mocilac *et al.*, 2018 ▸), are similar to (I) and (II). MEYYUK, an *N*-anthracene-9-carboxamide derivative (Kohmoto *et al.*, 2001 ▸) and MOCTUT or *N*,*N*-dibenzoyl-4-chloro­aniline structures (Usman *et al.*, 2002 ▸) are also similar in structure and conformation.

Shukla and co-workers have detailed six halogenated *N*-benzoyl-*N*-phenyl­benzamides (imides) that adopt both *syn* and *anti* conformations in the solid state (Shukla *et al.*, 2018 ▸). The reason why they adopt either conformation is not obvious and suggests that a transformation between either conformation as having a low activation energy barrier. Such imide behaviour (in adopting either of the *syn* or *anti* structures) has been known for decades although there does not seem to have been much investigation into possible fluxional behaviour and various influences driving towards one particular conformation or other.

## Synthesis and crystallization:   

Compound (I) is **Brmod** and (II) is **Brmopzd**. (I) and (II) were synthesized as mixtures together with the (1:1) benzamides and separated from the benzamides by standard column chromatography in good yields.

(I): Yield = 30–40% ^1^H NMR (CDCl_3_) for (I) with *J* values in Hz: δ 7.10 (1H, *dd*, ^3^ *J* = 7.5, ^4^ *J* = 5, ^5^ *J* = 1), 7.29 (1H, *t*, ^3^ *J* = 7.8), 7.33 (1H, *t*, ^3^ *J* = 7.9), 7.65 (2H, *dq*, ^3^ *J* = 8.4, ^4^ *J* = 1.8, ^5^ *J* = 1), 7.78 (1H, *ddd*, ^3^ *J* = 8, ^4^ *J* = 2, ^5^ *J* = 1), 7.90 (1H, *dt*, ^3^ *J* = 8, ^4^ *J* = 1), 7.98 (1H, *dt*, ^3^ *J* = 7.8, ^4^ *J* = 1), 8.17 (1H, *dd*, ^3^ *J* = 1.7), 8.21 (2H, *dd*, ^3^ *J* = 5.2, ^4^ *J* = 1), 8.40 (1H, *d*, ^3^ *J* = 8.5). IR (ATR): 2921 (*m*), 1683 (*s*), 1580 (*m*). Melting point 418–420 K.

(II): Yield = 45–55%. ^1^H NMR (CDCl_3_) for (I) with *J* values in Hz: δ 7.12 (1H, *t*, ^3^ *J* = 4.9), 7.18 (2H, *t*, ^3^ *J* = 12), 7.56 (2H, *ddd*, ^3^ *J* = 8.0, ^4^ *J* = 2.0, ^5^ *J* = 1.0), 7.60 (2H, *ddd*, ^3^ *J* = 7.8, ^4^ *J* = 1.7, ^5^ *J* = 1.0), 7.88 (2H, *t*, ^4^ *J* = 1.6), 8.59 (2H, *d*, ^3^ *J* = 4.8). IR (ATR): 3072 (*s*), 2963 (*s*), 1719 (*s*), 1682 (*m*). Melting point 406–411 K.

## Refinement   

Crystal data, data collection and structure refinement details are summarized in Table 3 ▸. H atoms attached to C atoms were treated as riding using the *SHELXL14/7* (Sheldrick, 2015*b* ▸) defaults at 294 (1) K with C—H = 0.93 Å (aromatic) and *U*_iso_(H) = 1.2*U*_eq_(C) (aromatic).

## Supplementary Material

Crystal structure: contains datablock(s) global, Brmod, Brmopzd. DOI: 10.1107/S2056989020014413/ex2039sup1.cif

Structure factors: contains datablock(s) Brmod. DOI: 10.1107/S2056989020014413/ex2039Brmodsup2.hkl

Supporting information file. DOI: 10.1107/S2056989020014413/ex2039Brmodsup4.cdx

Structure factors: contains datablock(s) Brmopzd. DOI: 10.1107/S2056989020014413/ex2039Brmopzdsup3.hkl

Supporting information file. DOI: 10.1107/S2056989020014413/ex2039Brmopzdsup5.cdx

Supporting information file. DOI: 10.1107/S2056989020014413/ex2039Brmodsup6.cml

Supporting information file. DOI: 10.1107/S2056989020014413/ex2039Brmopzdsup7.cml

CCDC references: 2041345, 2041344

Additional supporting information:  crystallographic information; 3D view; checkCIF report

## Figures and Tables

**Figure 2:**
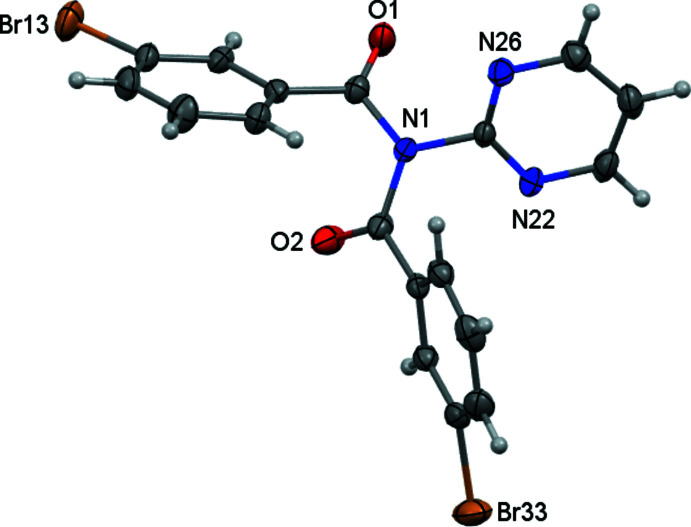
An *ORTEP* view of (II) with the atomic numbering scheme. Displacement ellipsoids are drawn at the 30% probability level.

**Figure 5:**
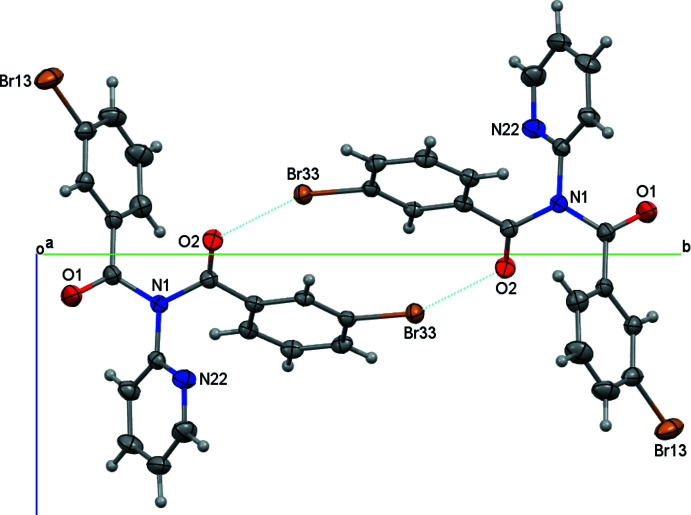
The inter­molecular inter­actions in (I) (C19 H12 Br2 N2 O2′a) with displacement ellipsoids at the 30% level.

**Figure 6:**
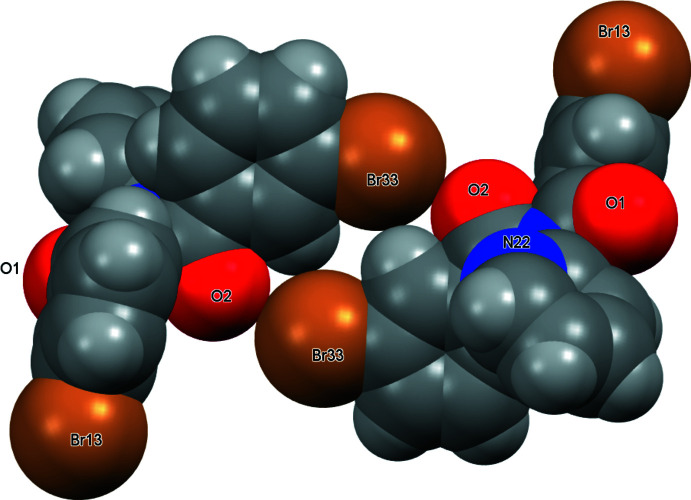
Inter­molecular inter­actions in (I) with atoms depicted as their van der Waals spheres.

**Figure 7:**
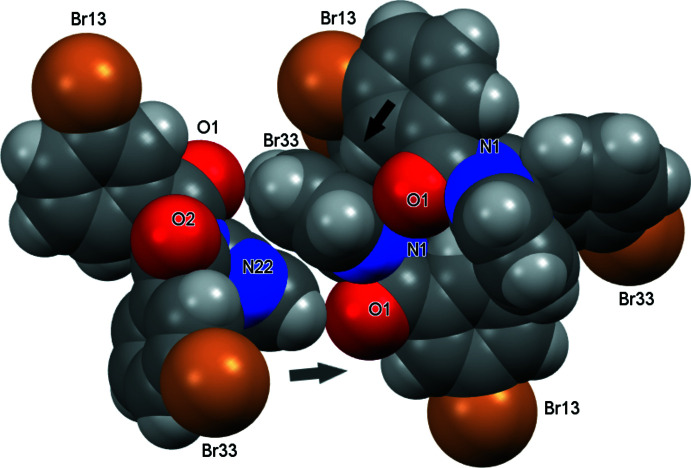
Inter­molecular inter­actions in (II) (shown with arrows) and with atoms depicted as their van der Waals spheres.

**Table 1:** Hydrogen-bond geometry (Å, °) for **Brmod** [Chem scheme1]

*D*—H⋯*A*	*D*—H	H⋯*A*	*D*⋯*A*	*D*—H⋯*A*
C12—H12⋯O1^i^	0.93	2.41	3.330 (4)	170
C32—H32⋯Br33^ii^	0.93	3.01	3.896 (3)	162
C36—H36⋯N22^iii^	0.93	2.68	3.363 (4)	131

Symmetry codes: (i) 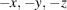; (ii) 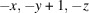; (iii) 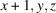.

**Table 2:** Hydrogen-bond geometry (Å, °) for **Brmopzd** [Chem scheme1]

*D*—H⋯*A*	*D*—H	H⋯*A*	*D*⋯*A*	*D*—H⋯*A*
C23—H23⋯O1^i^	0.93	2.65	3.369 (5)	134
C36—H36⋯O2^ii^	0.93	2.61	3.375 (5)	140
C12—H12⋯C25^iii^	0.93	2.76	3.677 (5)	168

Symmetry codes: (i) 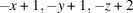; (ii) 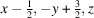; (iii) 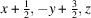.

**Table 3:** Experimental details

	**Brmod**	**Brmopzd**
Crystal data		
Chemical formula	C_19_H_12_Br_2_N_2_O_2_	C_18_H_11_Br_2_N_3_O_2_
*M*_r_	460.13	461.12
Crystal system, space group	Monoclinic, *P*2_1_/*c*	Monoclinic, *P*2_1_/*a*
Temperature (K)	294	294
*a*, *b*, *c* (Å)	5.5439 (1), 16.3366 (4), 19.3701 (4)	11.1712 (4), 11.0590 (3), 14.4181 (5)
β (°)	91.459 (2)	102.756 (4)
*V* (Å^3^)	1753.75 (6)	1737.28 (10)
*Z*	4	4
Radiation type	Mo *K*α	Mo *K*α
μ (mm^−1^)	4.64	4.68
Crystal size (mm)	0.43 × 0.35 × 0.18	0.22 × 0.20 × 0.05

Data collection		
Diffractometer	Rigaku Xcalibur, Sapphire3, Gemini Ultra	Rigaku Xcalibur, Sapphire3, Gemini Ultra
Absorption correction	Analytical (*ABSFAC*; Clark & Reid, 1998 ▸)	Analytical (*ABSFAC*; Clark & Reid, 1998 ▸)
*T*_min_, *T*_max_	0.228, 0.493	0.425, 0.801
No. of measured, independent and observed [*I* > 2σ(*I*)] reflections	16613, 4665, 3025	13616, 3865, 2219
*R*_int_	0.037	0.047
(sin θ/λ)_max_ (Å^−1^)	0.694	0.657

Refinement		
*R*[*F* ^2^ > 2σ(*F* ^2^)], *wR*(*F* ^2^), *S*	0.042, 0.085, 1.01	0.052, 0.109, 1.02
No. of reflections	4665	3865
No. of parameters	226	226
H-atom treatment	H-atom parameters constrained	H-atom parameters constrained
Δρ_max_, Δρ_min_ (e Å^−3^)	0.60, −0.42	0.89, −0.67

Computer programs: *CrysAlis PRO* (Rigaku OD, 2015 ▸), *SHELXT14/7* (Sheldrick, 2015*a* ▸), *SHELXL14/7* (Sheldrick, 2015*b* ▸) and *Mercury* (Macrae *et al.*, 2020 ▸).

## supplementary crystallographic information

### 3-Bromo-N-(3-bromobenzoyl)-N-(pyridin-2-yl)benzamide (Brmod) Crystal data

**Table:**

C_19_H_12_Br_2_N_2_O_2_	*D*_x_ = 1.743 Mg m^−^^3^
*M**_r_* = 460.13	Melting point: 419 K
Monoclinic, *P*2_1_/*c*	Mo *K*α radiation, λ = 0.71073 Å
*a* = 5.5439 (1) Å	Cell parameters from 4432 reflections
*b* = 16.3366 (4) Å	θ = 2.1–29.5°
*c* = 19.3701 (4) Å	µ = 4.64 mm^−^^1^
β = 91.459 (2)°	*T* = 294 K
*V* = 1753.75 (6) Å^3^	Block, colourless
*Z* = 4	0.43 × 0.35 × 0.18 mm
*F*(000) = 904	

### 3-Bromo-N-(3-bromobenzoyl)-N-(pyridin-2-yl)benzamide (Brmod) Data collection

**Table:**

Rigaku Xcalibur, Sapphire3, Gemini Ultra diffractometer	4665 independent reflections
Radiation source: Enhance (Mo) X-ray Source	3025 reflections with *I* > 2σ(*I*)
Graphite monochromator	*R*_int_ = 0.037
Detector resolution: 16.0560 pixels mm^-1^	θ_max_ = 29.6°, θ_min_ = 2.1°
ω scans	*h* = −7→7
Absorption correction: analytical (*ABSFAC*; Clark & Reid, 1998)	*k* = −22→17
*T*_min_ = 0.228, *T*_max_ = 0.493	*l* = −26→26
16613 measured reflections	

### 3-Bromo-N-(3-bromobenzoyl)-N-(pyridin-2-yl)benzamide (Brmod) Refinement

**Table:**

Refinement on *F*^2^	Primary atom site location: structure-invariant direct methods
Least-squares matrix: full	Secondary atom site location: difference Fourier map
*R*[*F*^2^ > 2σ(*F*^2^)] = 0.042	Hydrogen site location: inferred from neighbouring sites
*wR*(*F*^2^) = 0.085	H-atom parameters constrained
*S* = 1.01	*w* = 1/[σ^2^(*F*_o_^2^) + (0.0295*P*)^2^ + 0.9875*P*] where *P* = (*F*_o_^2^ + 2*F*_c_^2^)/3
4665 reflections	(Δ/σ)_max_ < 0.001
226 parameters	Δρ_max_ = 0.60 e Å^−^^3^
0 restraints	Δρ_min_ = −0.42 e Å^−^^3^

### 3-Bromo-N-(3-bromobenzoyl)-N-(pyridin-2-yl)benzamide (Brmod) Special details

**Table:**

Geometry. All esds (except the esd in the dihedral angle between two l.s. planes) are estimated using the full covariance matrix. The cell esds are taken into account individually in the estimation of esds in distances, angles and torsion angles; correlations between esds in cell parameters are only used when they are defined by crystal symmetry. An approximate (isotropic) treatment of cell esds is used for estimating esds involving l.s. planes.

### 3-Bromo-N-(3-bromobenzoyl)-N-(pyridin-2-yl)benzamide (Brmod) Fractional atomic coordinates and isotropic or equivalent isotropic displacement parameters (Å^2^)

**Table:**

	*x*	*y*	*z*	*U*_iso_*/*U*_eq_	
Br13	0.12586 (8)	0.01924 (3)	−0.22896 (2)	0.07723 (16)	
Br33	0.25059 (6)	0.58666 (2)	0.07841 (2)	0.04753 (11)	
O1	0.2102 (5)	0.05436 (15)	0.05540 (13)	0.0741 (8)	
C1	0.2745 (6)	0.11718 (19)	0.02838 (16)	0.0432 (7)	
N1	0.2894 (4)	0.19022 (14)	0.06634 (12)	0.0362 (5)	
O2	0.1237 (4)	0.27325 (13)	−0.01771 (11)	0.0513 (6)	
C2	0.2485 (5)	0.26745 (17)	0.03375 (15)	0.0350 (6)	
C11	0.3581 (5)	0.11967 (17)	−0.04341 (15)	0.0393 (7)	
C12	0.2308 (6)	0.07512 (18)	−0.09348 (15)	0.0432 (7)	
H12	0.0952	0.0451	−0.0820	0.052*	
C13	0.3084 (6)	0.0762 (2)	−0.15995 (16)	0.0499 (8)	
C14	0.5141 (7)	0.1177 (3)	−0.17788 (19)	0.0648 (10)	
H14	0.5647	0.1176	−0.2233	0.078*	
C15	0.6432 (7)	0.1592 (3)	−0.1273 (2)	0.0673 (11)	
H15	0.7851	0.1859	−0.1384	0.081*	
C16	0.5651 (6)	0.1615 (2)	−0.06085 (18)	0.0510 (8)	
H16	0.6509	0.1912	−0.0274	0.061*	
C21	0.2667 (5)	0.18650 (17)	0.13984 (14)	0.0366 (6)	
N22	0.0859 (4)	0.22893 (17)	0.16417 (13)	0.0488 (7)	
C23	0.0675 (7)	0.2294 (2)	0.23268 (19)	0.0626 (10)	
H23	−0.0594	0.2586	0.2513	0.075*	
C24	0.2231 (7)	0.1898 (2)	0.27726 (18)	0.0601 (9)	
H24	0.2045	0.1929	0.3248	0.072*	
C25	0.4070 (7)	0.1455 (2)	0.24981 (18)	0.0601 (9)	
H25	0.5145	0.1170	0.2785	0.072*	
C26	0.4310 (6)	0.1437 (2)	0.17975 (16)	0.0494 (8)	
H26	0.5550	0.1144	0.1598	0.059*	
C31	0.3759 (5)	0.33794 (17)	0.06655 (13)	0.0321 (6)	
C32	0.2767 (5)	0.41557 (17)	0.05842 (14)	0.0330 (6)	
H32	0.1309	0.4226	0.0344	0.040*	
C33	0.3957 (5)	0.48156 (17)	0.08614 (14)	0.0338 (6)	
C34	0.6181 (5)	0.47370 (19)	0.11981 (15)	0.0405 (7)	
H34	0.6988	0.5193	0.1375	0.049*	
C35	0.7172 (5)	0.39622 (19)	0.12645 (15)	0.0399 (7)	
H35	0.8668	0.3898	0.1486	0.048*	
C36	0.5982 (5)	0.32877 (17)	0.10082 (14)	0.0352 (6)	
H36	0.6657	0.2770	0.1063	0.042*	

### 3-Bromo-N-(3-bromobenzoyl)-N-(pyridin-2-yl)benzamide (Brmod) Atomic displacement parameters (Å^2^)

**Table:**

	*U*^11^	*U*^22^	*U*^33^	*U*^12^	*U*^13^	*U*^23^
Br13	0.0878 (3)	0.0939 (3)	0.0490 (2)	0.0319 (2)	−0.0185 (2)	−0.0193 (2)
Br33	0.0583 (2)	0.03904 (17)	0.04500 (18)	0.00725 (15)	−0.00459 (14)	−0.00051 (14)
O1	0.120 (2)	0.0505 (14)	0.0518 (15)	−0.0332 (15)	0.0154 (15)	0.0002 (12)
C1	0.0492 (19)	0.0393 (17)	0.0412 (17)	−0.0064 (14)	0.0016 (14)	0.0031 (14)
N1	0.0388 (14)	0.0361 (13)	0.0336 (13)	0.0011 (10)	0.0005 (10)	0.0022 (10)
O2	0.0563 (13)	0.0443 (12)	0.0518 (13)	0.0085 (10)	−0.0264 (11)	−0.0012 (10)
C2	0.0307 (15)	0.0375 (16)	0.0365 (16)	0.0056 (12)	−0.0020 (12)	0.0006 (13)
C11	0.0455 (17)	0.0330 (15)	0.0396 (17)	0.0059 (13)	0.0039 (13)	0.0023 (13)
C12	0.0483 (18)	0.0386 (17)	0.0428 (18)	0.0066 (14)	0.0017 (14)	−0.0007 (14)
C13	0.058 (2)	0.053 (2)	0.0384 (17)	0.0204 (16)	−0.0013 (15)	−0.0026 (15)
C14	0.064 (2)	0.087 (3)	0.044 (2)	0.019 (2)	0.0157 (18)	0.005 (2)
C15	0.052 (2)	0.087 (3)	0.063 (3)	0.002 (2)	0.0215 (19)	0.010 (2)
C16	0.0432 (19)	0.053 (2)	0.057 (2)	0.0008 (15)	0.0044 (16)	0.0014 (17)
C21	0.0359 (16)	0.0377 (16)	0.0362 (16)	−0.0010 (12)	−0.0007 (12)	0.0025 (13)
N22	0.0435 (15)	0.0615 (18)	0.0418 (15)	0.0131 (13)	0.0082 (12)	0.0034 (13)
C23	0.064 (2)	0.071 (3)	0.054 (2)	0.0150 (19)	0.0177 (19)	−0.0005 (19)
C24	0.079 (3)	0.066 (2)	0.0359 (18)	−0.006 (2)	0.0035 (18)	0.0029 (17)
C25	0.064 (2)	0.072 (2)	0.0439 (19)	0.0064 (19)	−0.0087 (17)	0.0174 (18)
C26	0.0498 (19)	0.054 (2)	0.0446 (18)	0.0146 (16)	0.0012 (15)	0.0084 (16)
C31	0.0269 (14)	0.0395 (15)	0.0299 (14)	0.0024 (12)	0.0010 (11)	0.0031 (12)
C32	0.0270 (13)	0.0423 (16)	0.0295 (13)	0.0029 (12)	−0.0021 (11)	0.0027 (12)
C33	0.0362 (16)	0.0372 (15)	0.0282 (14)	0.0031 (12)	0.0043 (12)	0.0022 (12)
C34	0.0346 (16)	0.0499 (18)	0.0368 (16)	−0.0065 (14)	−0.0015 (12)	−0.0045 (14)
C35	0.0251 (14)	0.057 (2)	0.0373 (16)	0.0006 (13)	−0.0039 (12)	0.0020 (14)
C36	0.0287 (14)	0.0418 (16)	0.0352 (15)	0.0047 (12)	0.0019 (12)	0.0038 (13)

### 3-Bromo-N-(3-bromobenzoyl)-N-(pyridin-2-yl)benzamide (Brmod) Geometric parameters (Å, º)

**Table:**

Br13—C13	1.900 (3)	C21—C26	1.371 (4)
Br33—C33	1.900 (3)	N22—C23	1.334 (4)
O1—C1	1.210 (4)	C23—C24	1.368 (5)
C1—N1	1.403 (4)	C23—H23	0.9300
C1—C11	1.478 (4)	C24—C25	1.368 (5)
N1—C2	1.426 (3)	C24—H24	0.9300
N1—C21	1.434 (3)	C25—C26	1.367 (4)
O2—C2	1.203 (3)	C25—H25	0.9300
C2—C31	1.485 (4)	C26—H26	0.9300
C11—C16	1.385 (4)	C31—C32	1.390 (4)
C11—C12	1.390 (4)	C31—C36	1.393 (4)
C12—C13	1.368 (4)	C32—C33	1.367 (4)
C12—H12	0.9300	C32—H32	0.9300
C13—C14	1.378 (5)	C33—C34	1.386 (4)
C14—C15	1.377 (5)	C34—C35	1.384 (4)
C14—H14	0.9300	C34—H34	0.9300
C15—C16	1.369 (5)	C35—C36	1.371 (4)
C15—H15	0.9300	C35—H35	0.9300
C16—H16	0.9300	C36—H36	0.9300
C21—N22	1.316 (4)		
			
O1—C1—N1	120.6 (3)	N22—C23—C24	124.2 (3)
O1—C1—C11	122.2 (3)	N22—C23—H23	117.9
N1—C1—C11	117.0 (3)	C24—C23—H23	117.9
C1—N1—C2	120.9 (2)	C23—C24—C25	118.0 (3)
C1—N1—C21	118.6 (2)	C23—C24—H24	121.0
C2—N1—C21	117.4 (2)	C25—C24—H24	121.0
O2—C2—N1	121.2 (3)	C26—C25—C24	119.3 (3)
O2—C2—C31	123.4 (3)	C26—C25—H25	120.3
N1—C2—C31	115.3 (2)	C24—C25—H25	120.3
C16—C11—C12	119.8 (3)	C25—C26—C21	117.9 (3)
C16—C11—C1	121.7 (3)	C25—C26—H26	121.0
C12—C11—C1	118.4 (3)	C21—C26—H26	121.0
C13—C12—C11	118.9 (3)	C32—C31—C36	119.7 (3)
C13—C12—H12	120.6	C32—C31—C2	118.5 (2)
C11—C12—H12	120.6	C36—C31—C2	121.7 (2)
C12—C13—C14	121.7 (3)	C33—C32—C31	119.3 (2)
C12—C13—Br13	118.8 (3)	C33—C32—H32	120.3
C14—C13—Br13	119.5 (3)	C31—C32—H32	120.3
C15—C14—C13	118.8 (3)	C32—C33—C34	121.8 (3)
C15—C14—H14	120.6	C32—C33—Br33	118.8 (2)
C13—C14—H14	120.6	C34—C33—Br33	119.4 (2)
C16—C15—C14	120.7 (3)	C35—C34—C33	118.3 (3)
C16—C15—H15	119.6	C35—C34—H34	120.8
C14—C15—H15	119.6	C33—C34—H34	120.8
C15—C16—C11	120.0 (3)	C36—C35—C34	121.0 (3)
C15—C16—H16	120.0	C36—C35—H35	119.5
C11—C16—H16	120.0	C34—C35—H35	119.5
N22—C21—C26	124.6 (3)	C35—C36—C31	119.9 (3)
N22—C21—N1	114.9 (2)	C35—C36—H36	120.1
C26—C21—N1	120.5 (3)	C31—C36—H36	120.1
C21—N22—C23	116.0 (3)		
			
O1—C1—N1—C2	−149.3 (3)	C1—N1—C21—C26	62.3 (4)
C11—C1—N1—C2	35.3 (4)	C2—N1—C21—C26	−137.4 (3)
O1—C1—N1—C21	10.3 (4)	C26—C21—N22—C23	0.4 (5)
C11—C1—N1—C21	−165.1 (2)	N1—C21—N22—C23	−177.1 (3)
C1—N1—C2—O2	26.1 (4)	C21—N22—C23—C24	0.5 (5)
C21—N1—C2—O2	−133.7 (3)	N22—C23—C24—C25	−1.3 (6)
C1—N1—C2—C31	−152.0 (3)	C23—C24—C25—C26	1.2 (6)
C21—N1—C2—C31	48.1 (3)	C24—C25—C26—C21	−0.4 (5)
O1—C1—C11—C16	−133.9 (3)	N22—C21—C26—C25	−0.4 (5)
N1—C1—C11—C16	41.4 (4)	N1—C21—C26—C25	176.9 (3)
O1—C1—C11—C12	42.8 (4)	O2—C2—C31—C32	28.3 (4)
N1—C1—C11—C12	−141.9 (3)	N1—C2—C31—C32	−153.6 (2)
C16—C11—C12—C13	−2.5 (4)	O2—C2—C31—C36	−147.3 (3)
C1—C11—C12—C13	−179.2 (3)	N1—C2—C31—C36	30.8 (4)
C11—C12—C13—C14	2.5 (5)	C36—C31—C32—C33	−2.0 (4)
C11—C12—C13—Br13	−177.3 (2)	C2—C31—C32—C33	−177.7 (2)
C12—C13—C14—C15	−0.3 (5)	C31—C32—C33—C34	2.7 (4)
Br13—C13—C14—C15	179.5 (3)	C31—C32—C33—Br33	−177.05 (19)
C13—C14—C15—C16	−2.1 (6)	C32—C33—C34—C35	−1.5 (4)
C14—C15—C16—C11	2.1 (6)	Br33—C33—C34—C35	178.3 (2)
C12—C11—C16—C15	0.2 (5)	C33—C34—C35—C36	−0.4 (4)
C1—C11—C16—C15	176.9 (3)	C34—C35—C36—C31	1.0 (4)
C1—N1—C21—N22	−120.1 (3)	C32—C31—C36—C35	0.2 (4)
C2—N1—C21—N22	40.3 (3)	C2—C31—C36—C35	175.7 (3)

### 3-Bromo-N-(3-bromobenzoyl)-N-(pyridin-2-yl)benzamide (Brmod) Hydrogen-bond geometry (Å, º)

**Table:**

*D*—H···*A*	*D*—H	H···*A*	*D*···*A*	*D*—H···*A*
C12—H12···O1^i^	0.93	2.41	3.330 (4)	170
C32—H32···Br33^ii^	0.93	3.01	3.896 (3)	162
C36—H36···N22^iii^	0.93	2.68	3.363 (4)	131

Symmetry codes: (i) −*x*, −*y*, −*z*; (ii) −*x*, −*y*+1, −*z*; (iii) *x*+1, *y*, *z*.

### 3-Bromo-N-(3-bromobenzoyl)-N-(pyrimidin-2-yl)benzamide (Brmopzd) Crystal data

**Table:**

C_18_H_11_Br_2_N_3_O_2_	*D*_x_ = 1.763 Mg m^−^^3^
*M**_r_* = 461.12	Melting point: 408 K
Monoclinic, *P*2_1_/*a*	Mo *K*α radiation, λ = 0.71073 Å
*a* = 11.1712 (4) Å	Cell parameters from 3165 reflections
*b* = 11.0590 (3) Å	θ = 3.2–27.8°
*c* = 14.4181 (5) Å	µ = 4.68 mm^−^^1^
β = 102.756 (4)°	*T* = 294 K
*V* = 1737.28 (10) Å^3^	Plate, colourless
*Z* = 4	0.22 × 0.20 × 0.05 mm
*F*(000) = 904	

### 3-Bromo-N-(3-bromobenzoyl)-N-(pyrimidin-2-yl)benzamide (Brmopzd) Data collection

**Table:**

Rigaku Xcalibur, Sapphire3, Gemini Ultra diffractometer	3865 independent reflections
Radiation source: Enhance (Mo) X-ray Source	2219 reflections with *I* > 2σ(*I*)
Detector resolution: 16.056 pixels mm^-1^	*R*_int_ = 0.047
ω scans	θ_max_ = 27.9°, θ_min_ = 3.2°
Absorption correction: analytical (*ABSFAC*; Clark & Reid, 1998)	*h* = −14→14
*T*_min_ = 0.425, *T*_max_ = 0.801	*k* = −14→13
13616 measured reflections	*l* = −18→15

### 3-Bromo-N-(3-bromobenzoyl)-N-(pyrimidin-2-yl)benzamide (Brmopzd) Refinement

**Table:**

Refinement on *F*^2^	Primary atom site location: structure-invariant direct methods
Least-squares matrix: full	Secondary atom site location: difference Fourier map
*R*[*F*^2^ > 2σ(*F*^2^)] = 0.052	Hydrogen site location: inferred from neighbouring sites
*wR*(*F*^2^) = 0.109	H-atom parameters constrained
*S* = 1.02	*w* = 1/[σ^2^(*F*_o_^2^) + (0.0345*P*)^2^ + 1.6333*P*] where *P* = (*F*_o_^2^ + 2*F*_c_^2^)/3
3865 reflections	(Δ/σ)_max_ < 0.001
226 parameters	Δρ_max_ = 0.89 e Å^−^^3^
0 restraints	Δρ_min_ = −0.67 e Å^−^^3^

### 3-Bromo-N-(3-bromobenzoyl)-N-(pyrimidin-2-yl)benzamide (Brmopzd) Special details

**Table:**

Geometry. All esds (except the esd in the dihedral angle between two l.s. planes) are estimated using the full covariance matrix. The cell esds are taken into account individually in the estimation of esds in distances, angles and torsion angles; correlations between esds in cell parameters are only used when they are defined by crystal symmetry. An approximate (isotropic) treatment of cell esds is used for estimating esds involving l.s. planes.

### 3-Bromo-N-(3-bromobenzoyl)-N-(pyrimidin-2-yl)benzamide (Brmopzd) Fractional atomic coordinates and isotropic or equivalent isotropic displacement parameters (Å^2^)

**Table:**

	*x*	*y*	*z*	*U*_iso_*/*U*_eq_	
Br13	0.56862 (6)	1.25414 (5)	0.79435 (5)	0.0721 (2)	
Br33	0.38119 (6)	0.32158 (5)	0.48956 (4)	0.0757 (2)	
O1	0.4800 (3)	0.8188 (3)	0.9503 (2)	0.0537 (9)	
C1	0.4245 (4)	0.8174 (4)	0.8691 (3)	0.0381 (10)	
N1	0.3759 (3)	0.7061 (3)	0.8250 (2)	0.0371 (9)	
O2	0.4771 (3)	0.7308 (3)	0.7062 (2)	0.0625 (10)	
C2	0.3990 (4)	0.6770 (4)	0.7352 (3)	0.0408 (11)	
C11	0.3956 (4)	0.9282 (4)	0.8105 (3)	0.0355 (10)	
C12	0.4786 (4)	1.0231 (4)	0.8278 (3)	0.0392 (11)	
H12	0.5518	1.0153	0.8730	0.047*	
C13	0.4518 (4)	1.1282 (4)	0.7777 (3)	0.0428 (11)	
C14	0.3428 (5)	1.1429 (4)	0.7130 (4)	0.0546 (13)	
H14	0.3251	1.2156	0.6804	0.066*	
C15	0.2597 (5)	1.0492 (5)	0.6966 (4)	0.0601 (14)	
H15	0.1853	1.0587	0.6531	0.072*	
C16	0.2864 (4)	0.9413 (4)	0.7445 (3)	0.0449 (12)	
H16	0.2309	0.8775	0.7324	0.054*	
C21	0.3492 (4)	0.6130 (3)	0.8853 (3)	0.0330 (10)	
N22	0.3981 (3)	0.5060 (3)	0.8759 (3)	0.0434 (9)	
C23	0.3708 (5)	0.4203 (4)	0.9333 (3)	0.0519 (13)	
H23	0.3987	0.3420	0.9278	0.062*	
C24	0.3035 (5)	0.4434 (4)	0.9998 (4)	0.0539 (13)	
H24	0.2883	0.3837	1.0412	0.065*	
C25	0.2596 (4)	0.5580 (4)	1.0027 (3)	0.0507 (12)	
H25	0.2134	0.5762	1.0473	0.061*	
N26	0.2803 (3)	0.6456 (3)	0.9440 (3)	0.0427 (9)	
C31	0.3208 (4)	0.5834 (3)	0.6777 (3)	0.0332 (10)	
C32	0.3740 (4)	0.5108 (4)	0.6202 (3)	0.0382 (10)	
H32	0.4567	0.5194	0.6199	0.046*	
C33	0.3045 (5)	0.4261 (4)	0.5636 (3)	0.0447 (12)	
C34	0.1809 (5)	0.4155 (5)	0.5591 (3)	0.0563 (14)	
H34	0.1339	0.3595	0.5186	0.068*	
C35	0.1276 (5)	0.4898 (5)	0.6159 (4)	0.0546 (13)	
H35	0.0439	0.4837	0.6132	0.066*	
C36	0.1967 (4)	0.5727 (4)	0.6763 (3)	0.0426 (11)	
H36	0.1606	0.6207	0.7156	0.051*	

### 3-Bromo-N-(3-bromobenzoyl)-N-(pyrimidin-2-yl)benzamide (Brmopzd) Atomic displacement parameters (Å^2^)

**Table:**

	*U*^11^	*U*^22^	*U*^33^	*U*^12^	*U*^13^	*U*^23^
Br13	0.0839 (4)	0.0390 (3)	0.0959 (5)	−0.0179 (3)	0.0255 (4)	0.0052 (3)
Br33	0.1132 (5)	0.0612 (4)	0.0532 (3)	0.0186 (3)	0.0194 (3)	−0.0170 (3)
O1	0.065 (2)	0.0410 (18)	0.049 (2)	−0.0105 (17)	−0.0011 (18)	0.0076 (16)
C1	0.037 (3)	0.034 (2)	0.044 (3)	−0.003 (2)	0.010 (2)	0.001 (2)
N1	0.049 (2)	0.0248 (17)	0.041 (2)	−0.0025 (16)	0.0176 (18)	−0.0013 (16)
O2	0.070 (2)	0.054 (2)	0.078 (3)	−0.0242 (18)	0.048 (2)	−0.0217 (18)
C2	0.043 (3)	0.032 (2)	0.054 (3)	−0.001 (2)	0.025 (2)	−0.002 (2)
C11	0.045 (3)	0.030 (2)	0.035 (2)	0.004 (2)	0.015 (2)	0.0014 (18)
C12	0.042 (3)	0.030 (2)	0.047 (3)	−0.001 (2)	0.011 (2)	−0.004 (2)
C13	0.053 (3)	0.029 (2)	0.051 (3)	0.000 (2)	0.021 (3)	0.004 (2)
C14	0.069 (4)	0.038 (3)	0.058 (3)	0.010 (3)	0.016 (3)	0.014 (2)
C15	0.053 (3)	0.052 (3)	0.068 (4)	0.007 (3)	−0.002 (3)	0.010 (3)
C16	0.049 (3)	0.033 (2)	0.052 (3)	0.000 (2)	0.010 (3)	0.004 (2)
C21	0.037 (3)	0.026 (2)	0.036 (2)	−0.0034 (18)	0.007 (2)	0.0040 (18)
N22	0.055 (3)	0.0293 (19)	0.046 (2)	0.0070 (17)	0.0122 (19)	0.0050 (17)
C23	0.064 (4)	0.030 (2)	0.055 (3)	0.002 (2)	0.000 (3)	0.008 (2)
C24	0.061 (3)	0.050 (3)	0.050 (3)	−0.009 (3)	0.012 (3)	0.015 (2)
C25	0.052 (3)	0.054 (3)	0.049 (3)	−0.005 (2)	0.017 (3)	0.005 (2)
N26	0.047 (2)	0.035 (2)	0.050 (2)	−0.0034 (17)	0.021 (2)	−0.0009 (18)
C31	0.036 (3)	0.031 (2)	0.035 (2)	0.0033 (19)	0.013 (2)	0.0061 (18)
C32	0.042 (3)	0.035 (2)	0.040 (3)	0.003 (2)	0.017 (2)	0.004 (2)
C33	0.063 (3)	0.040 (3)	0.029 (3)	0.006 (2)	0.006 (2)	0.003 (2)
C34	0.068 (4)	0.054 (3)	0.041 (3)	−0.010 (3)	−0.002 (3)	0.002 (2)
C35	0.041 (3)	0.070 (4)	0.049 (3)	−0.007 (3)	0.002 (3)	0.016 (3)
C36	0.046 (3)	0.045 (3)	0.040 (3)	0.005 (2)	0.017 (2)	0.006 (2)

### 3-Bromo-N-(3-bromobenzoyl)-N-(pyrimidin-2-yl)benzamide (Brmopzd) Geometric parameters (Å, º)

**Table:**

Br13—C13	1.887 (4)	C21—N26	1.314 (5)
Br33—C33	1.901 (4)	C21—N22	1.323 (5)
O1—C1	1.199 (5)	N22—C23	1.337 (5)
C1—N1	1.435 (5)	C23—C24	1.367 (6)
C1—C11	1.483 (6)	C23—H23	0.9300
N1—C2	1.413 (5)	C24—C25	1.363 (6)
N1—C21	1.421 (5)	C24—H24	0.9300
O2—C2	1.204 (5)	C25—N26	1.340 (5)
C2—C31	1.483 (6)	C25—H25	0.9300
C11—C16	1.379 (6)	C31—C32	1.381 (5)
C11—C12	1.386 (6)	C31—C36	1.388 (6)
C12—C13	1.366 (6)	C32—C33	1.365 (6)
C12—H12	0.9300	C32—H32	0.9300
C13—C14	1.370 (7)	C33—C34	1.373 (7)
C14—C15	1.376 (7)	C34—C35	1.384 (7)
C14—H14	0.9300	C34—H34	0.9300
C15—C16	1.377 (6)	C35—C36	1.378 (6)
C15—H15	0.9300	C35—H35	0.9300
C16—H16	0.9300	C36—H36	0.9300
			
O1—C1—N1	120.5 (4)	C21—N22—C23	114.5 (4)
O1—C1—C11	123.1 (4)	N22—C23—C24	122.5 (4)
N1—C1—C11	116.3 (4)	N22—C23—H23	118.7
C2—N1—C21	120.1 (3)	C24—C23—H23	118.7
C2—N1—C1	118.2 (3)	C25—C24—C23	116.9 (4)
C21—N1—C1	117.4 (3)	C25—C24—H24	121.5
O2—C2—N1	119.9 (4)	C23—C24—H24	121.5
O2—C2—C31	122.2 (4)	N26—C25—C24	122.7 (5)
N1—C2—C31	117.8 (4)	N26—C25—H25	118.7
C16—C11—C12	119.9 (4)	C24—C25—H25	118.7
C16—C11—C1	121.9 (4)	C21—N26—C25	114.6 (4)
C12—C11—C1	118.1 (4)	C32—C31—C36	120.2 (4)
C13—C12—C11	119.3 (4)	C32—C31—C2	117.6 (4)
C13—C12—H12	120.4	C36—C31—C2	122.1 (4)
C11—C12—H12	120.4	C33—C32—C31	119.7 (4)
C12—C13—C14	121.3 (4)	C33—C32—H32	120.2
C12—C13—Br13	119.7 (4)	C31—C32—H32	120.2
C14—C13—Br13	119.0 (3)	C32—C33—C34	121.3 (4)
C13—C14—C15	119.5 (4)	C32—C33—Br33	119.0 (4)
C13—C14—H14	120.3	C34—C33—Br33	119.6 (4)
C15—C14—H14	120.3	C33—C34—C35	118.7 (5)
C14—C15—C16	120.2 (5)	C33—C34—H34	120.6
C14—C15—H15	119.9	C35—C34—H34	120.6
C16—C15—H15	119.9	C36—C35—C34	121.0 (5)
C15—C16—C11	119.8 (4)	C36—C35—H35	119.5
C15—C16—H16	120.1	C34—C35—H35	119.5
C11—C16—H16	120.1	C35—C36—C31	119.0 (4)
N26—C21—N22	128.7 (4)	C35—C36—H36	120.5
N26—C21—N1	115.3 (4)	C31—C36—H36	120.5
N22—C21—N1	116.0 (4)		
			
O1—C1—N1—C2	−131.1 (4)	C2—N1—C21—N22	28.9 (6)
C11—C1—N1—C2	52.3 (5)	C1—N1—C21—N22	−127.6 (4)
O1—C1—N1—C21	25.8 (6)	N26—C21—N22—C23	1.3 (7)
C11—C1—N1—C21	−150.8 (4)	N1—C21—N22—C23	−179.7 (4)
C21—N1—C2—O2	−141.4 (4)	C21—N22—C23—C24	−3.4 (7)
C1—N1—C2—O2	14.9 (6)	N22—C23—C24—C25	2.9 (7)
C21—N1—C2—C31	41.3 (6)	C23—C24—C25—N26	−0.1 (8)
C1—N1—C2—C31	−162.5 (4)	N22—C21—N26—C25	1.3 (7)
O1—C1—C11—C16	−143.6 (5)	N1—C21—N26—C25	−177.7 (4)
N1—C1—C11—C16	32.9 (6)	C24—C25—N26—C21	−1.9 (7)
O1—C1—C11—C12	32.4 (6)	O2—C2—C31—C32	35.6 (6)
N1—C1—C11—C12	−151.1 (4)	N1—C2—C31—C32	−147.1 (4)
C16—C11—C12—C13	−1.1 (6)	O2—C2—C31—C36	−140.4 (5)
C1—C11—C12—C13	−177.2 (4)	N1—C2—C31—C36	36.9 (6)
C11—C12—C13—C14	2.1 (7)	C36—C31—C32—C33	−1.7 (6)
C11—C12—C13—Br13	−175.8 (3)	C2—C31—C32—C33	−177.8 (4)
C12—C13—C14—C15	−1.4 (7)	C31—C32—C33—C34	3.4 (7)
Br13—C13—C14—C15	176.6 (4)	C31—C32—C33—Br33	−176.9 (3)
C13—C14—C15—C16	−0.4 (8)	C32—C33—C34—C35	−2.3 (7)
C14—C15—C16—C11	1.4 (7)	Br33—C33—C34—C35	177.9 (3)
C12—C11—C16—C15	−0.6 (6)	C33—C34—C35—C36	−0.3 (7)
C1—C11—C16—C15	175.3 (4)	C34—C35—C36—C31	1.8 (7)
C2—N1—C21—N26	−152.0 (4)	C32—C31—C36—C35	−0.8 (6)
C1—N1—C21—N26	51.6 (5)	C2—C31—C36—C35	175.1 (4)

### 3-Bromo-N-(3-bromobenzoyl)-N-(pyrimidin-2-yl)benzamide (Brmopzd) Hydrogen-bond geometry (Å, º)

**Table:**

*D*—H···*A*	*D*—H	H···*A*	*D*···*A*	*D*—H···*A*
C23—H23···O1^i^	0.93	2.65	3.369 (5)	134
C36—H36···O2^ii^	0.93	2.61	3.375 (5)	140
C12—H12···C25^iii^	0.93	2.76	3.677 (5)	168

Symmetry codes: (i) −*x*+1, −*y*+1, −*z*+2; (ii) *x*−1/2, −*y*+3/2, *z*; (iii) *x*+1/2, −*y*+3/2, *z*.
